# A Smartphone App to Manage Cirrhotic Ascites Among Outpatients: Feasibility Study

**DOI:** 10.2196/17770

**Published:** 2020-09-02

**Authors:** Patricia Bloom, Thomas Wang, Madeline Marx, Michelle Tagerman, Bradley Green, Ashwini Arvind, Jasmine Ha, Judith Bloom, James M Richter

**Affiliations:** 1 Department of Gastroenterology Massachusetts General Hospital Boston, MA United States; 2 Department of Medicine Massachusetts General Hospital Boston, MA United States

**Keywords:** health care innovation, ascites, telemedicine, health care delivery, technology, mobile phone

## Abstract

**Background:**

Ascites is a common, painful, and serious complication of cirrhosis. Body weight is a reliable proxy for ascites volume; therefore, daily weight monitoring is recommended to optimize ascites management.

**Objective:**

This study aims to evaluate the feasibility of a smartphone app in facilitating outpatient ascites management.

**Methods:**

In this feasibility study, patients with cirrhotic ascites requiring active management were identified in both inpatient and outpatient settings. Patients were provided with a Bluetooth-connected scale, which transmitted weight data to a smartphone app and then via the internet to an electronic medical record (EMR). Weights were monitored every weekday. In the event of a weight change of ≥5 lbs in 1 week, patients were called and administered a short symptom questionnaire, and providers received an email alert. The primary outcomes of this study were the percentage of enrolled days during which weight data were successfully transmitted to an EMR and the percentage of weight alerts that prompted responses by the provider.

**Results:**

In this study, 25 patients were enrolled: 12 (48%) were male, and the mean age was 58 (SD 13; range 35-81) years. A total of 18 (72%) inpatients were enrolled. Weight data were successfully transmitted to an EMR during 71.2% (697/979) of the study enrollment days, with technology issues reported on 16.5% (162/979) of the days. Of a total of 79 weight change alerts fired, 41 (52%) were triggered by weight loss and 38 (48%) were by weight gain. Providers responded in some fashion to 66 (84%) of the weight alerts and intervened in response to 45 (57%) of the alerts, for example, by contacting the patient, scheduling clinic or paracentesis appointments, modifying the diuretic dose, or requesting a laboratory workup. Providers responded equally to weight increase and decrease alerts (*P*=.87). The staff called patients a mean of 3.7 (SD 3.5) times per patient, and the number of phone calls correlated with technology issues (*r*=0.60; *P*=.002). A total of 60% (15/25) of the patients chose to extend their participation beyond 30 days. A total of 17 patient readmissions occurred during the study period, with only 4 (24%) related to ascites.

**Conclusions:**

We demonstrated the feasibility of a smartphone app to facilitate the management of ascites and reported excellent rates of patient and provider engagement. This innovation could enable early therapeutic intervention, thereby decreasing the burden of morbidity and mortality among patients with cirrhosis.

## Introduction

Ascites represents a major burden for patients with cirrhosis. Cirrhotic ascites is associated with poor health-related quality of life [1], hospital admissions [2-4], high cost of care [4-6], and increased mortality [3,7]. For decades, body weight has been identified as a useful proxy for ascites volume, but accurate weight monitoring at home has been difficult. In fact, monitoring weight is central to expert guidelines for ascites management [8,9] and treatment trials [10-12]. Weight changes signal a change in ascites volume and may provoke laboratory testing for renal injury, modification of diuretic dose, and large-volume paracentesis (LVP). Failure to recognize early signs of increasing ascites or overdiuresis has long been recognized as a preventable cause of ascites-related readmissions [6]. Timely transmission of accurate weight data from patients to their hepatology providers may allow for early intervention and prevent readmissions.

Technology represents a promising tool to facilitate the management of ascites by increasing the quality and quantity of patient-provider communication about weight data. In a recent interview study of patients with an early readmission for decompensated cirrhosis, the majority stated that they would use a smartphone to manage their condition, particularly if it was able to transmit weight data to their provider [13]. Retrospective and survey studies suggest that programs with enhanced outpatient care can improve outcomes for patients with ascites [14,15].

We created a simple telemonitoring program, in which patient weight data are communicated daily to an electronic medical record (EMR) via a Bluetooth-connected scale and a smartphone app, and alerts for significant weight changes are emailed to the hepatology provider. In this study, we assessed the feasibility of the telemonitoring program for ascites management. Specifically, we evaluated whether patients would regularly weigh themselves and whether providers would respond to weight alerts.

## Methods

### Study Design

**Figure 1:**
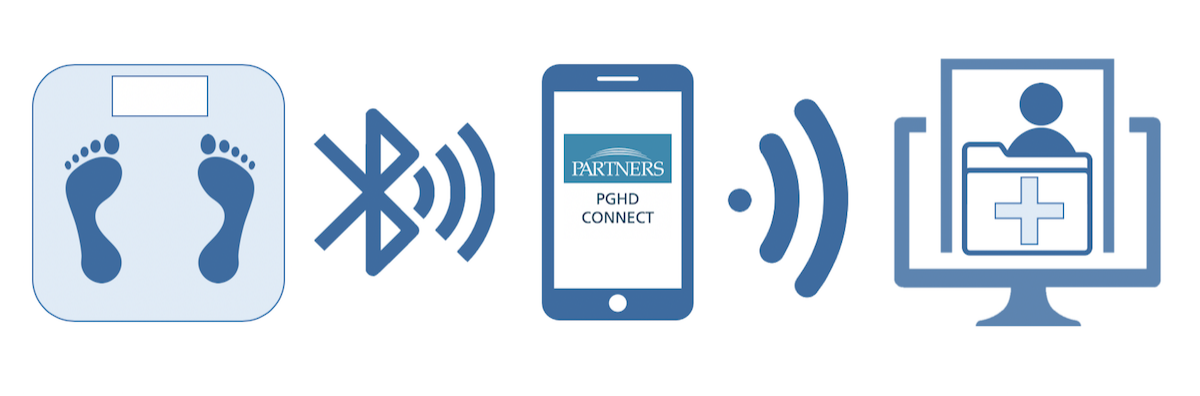
Flow of weight information. Weight data are collected from the Bluetooth-connected scale, transmitted via a Bluetooth connection to the PGHDConnect app, and then via the internet to the electronic medical record.

We conducted a feasibility study of an outpatient weight monitoring program for patients requiring active management of their cirrhotic ascites. Patients were consented, instructed in the use of the app, and provided with a Bluetooth-connected scale to use at home (Figure 1), which transmitted weight data to a smartphone app and then to the EMR. Weights were monitored every weekday, and significant weight changes prompted an email alert to hepatology providers. At the end of enrollment, patients, caregivers, and hepatology providers were interviewed for feedback. Written informed consent was obtained from patients, and a study fact sheet was given to caregivers and hepatology providers, who provided verbal consent to participate. This study was approved by the Partners HealthCare Institutional Review Board.

### The Technology

Eligible patients were assisted in downloading and registering for a no-cost smartphone app and were given a no-cost Bluetooth-connected scale (A&D UC-352BLE digital scale). Digital scale weights were transmitted via a Bluetooth connection to the Partners Patient-Generated Health Data Connect (PGHD*Connect*) app and then transmitted securely via the internet to the Partners *e*Care EMR (Epic). The PGHD*Connect* app is currently used for clinical care at Partners HealthCare and has been deemed Health Insurance Portability and Accountability Act (HIPAA)–compliant and secure by the Partners HealthCare information services team.

### Study Population

We enrolled patients receiving active management of their cirrhotic ascites, as this population may benefit most from an outpatient weight monitoring system. First, we performed daily screening of the inpatient hepatology consult census to identify patients with a clinical diagnosis of cirrhosis and requiring active management of ascites during their admission, including therapeutic paracentesis, diuretic hold or titration, or treatment of renal or electrolyte dysfunction resulting from ascites management. After several months, when hepatology providers were increasingly aware of the study, we began enrolling both inpatient and outpatient referrals from hepatology providers and stopped active screening of the inpatient census.

Patients were approached for consent and enrollment if deemed appropriate by their primary outpatient hepatology provider. Of importance, patients were required to own a smartphone and be able to stand for daily weighing to enroll, as these are essential requirements for the program to function. The diagnosis of cirrhosis and ascites was confirmed by the hepatology provider. Patients with poorly controlled hepatic encephalopathy and severe ongoing cognitive dysfunction were excluded. Other inclusion criteria included aged 18 years or older, English speaking, and capacity to provide informed consent.

Upon enrollment, patients were asked if they had a caregiver who would likely assist them in using the app and scale. A study fact sheet was provided to the patient, caregiver, and hepatology providers.

### Study Procedures

Once qualified patients provided informed consent, the study staff assisted them in downloading the PGHD*Connect* app and pairing the app with their scale. Nonphysician study staff (MM, MT, BD, and AA) monitored the patients’ weights daily in the EMR. In the event of a weight change of >5 lbs in 1 week, these study staff called and administered a short symptom questionnaire. The study staff then emailed providers a summary of the weight change event and answers to the short symptom questionnaire. Study staff also called patients if no weight data appeared in the EMR and troubleshot technology issues. Finally, the study staff tracked providers’ responses to weight change alerts. Weights were monitored every weekday for 4 weeks, though patients were allowed to enroll for shorter or longer durations. The program was paused during hospitalizations and resumed upon discharge.

Patients were enrolled between January and October 2019. At the end of the enrollment, a semistructured interview with patients, caregivers, and hepatology providers was conducted to obtain qualitative data through open-ended questions. Study staff (MM, MT, BG, and AA) audio-recorded an exit interview with the patients and, if available, their caregivers. A physician investigator (PB) performed all exit interviews with hepatology providers. Chart review was performed on all enrolled patients to obtain the following data: demographic factors, etiology and severity of liver disease, and diagnoses at index admission and readmission.

### Statistical Analysis

The primary outcomes of this study were the percentage of enrolled days during which weight data were successfully transmitted to the EMR and the percentage of weight alerts that prompted a response by the provider. Our team predetermined that receiving weight data in the EMR on 50% of the days enrolled would signal feasibility, as weight data are not required every single day for optimal ascites management. In addition, we determined that providers responding to at least 50% of weight alerts would constitute an adequate threshold for feasibility.

Descriptive data were summarized as mean (SD; continuous) or presented as proportions (categorical). Missing data were accounted for by adjusting the denominator. Significance testing was performed using the Student *t* test for continuous variables and Fisher exact test for categorical variables.

### Exit Interview

The purpose of the exit interviews was to evaluate facilitators and barriers to the intervention, to explore the root causes of outpatient ascites management failures, and to explore the desired features of a digital ascites management tool. We created a semistructured interview to target these themes for patients, caregivers, and hepatology providers (Multimedia Appendix 1). The interviewees were asked open-ended questions and were asked for further clarification when needed. The themes were generated based on the principle of qualitative research reflexivity: by reflecting on clinical experience, literature review, and preconceptions to reduce bias in interviewing and analysis [16]. The exit interviews were piloted on the authors and edited in an iterative fashion.

### Qualitative Data Analysis

Interview transcripts were imported into NVivo 11.0 (QSR International). Two investigators (PB and TW) iteratively read and coded interview transcripts for themes [17]. The principle of grounded theory was applied: as themes emerged from the data, specific lines of text were coded into themes [18]. The analysts then jointly compared codes, resolved discrepancies, and developed a taxonomy of themes. Themes were refined until saturation was reached, with a final taxonomy of 13 themes. This final taxonomy was applied to all transcripts by the 2 analysts, with a kappa agreement of 84%.

### Institutional Structure

This study was conducted at a single urban academic liver transplant center. Patients with cirrhosis were admitted to a hospital medicine service with hepatology or gastroenterology consultation. The majority of the patients were locals and lived within an hour’s drive from the hospital, although some patients were transferred from other parts of New England.

## Results

**Patient Characteristics**

**Figure 2:**
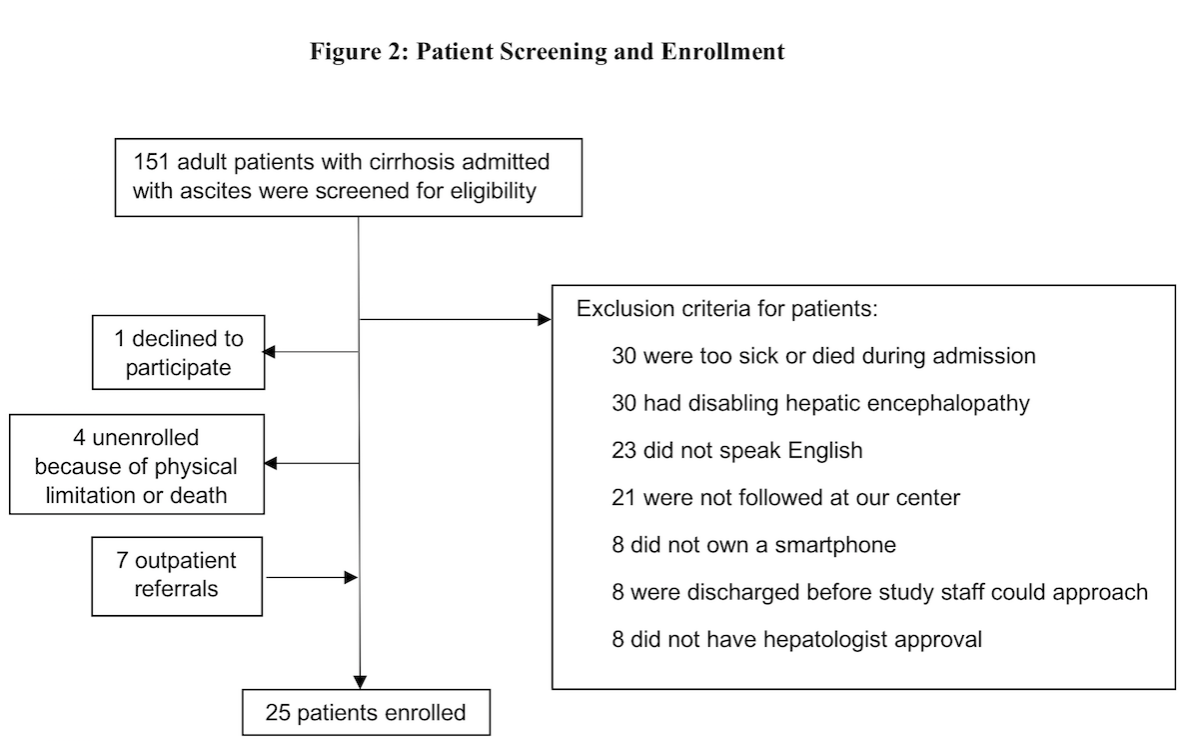
Patient screening flowchart.

After screening 151 consecutive adult patients admitted with cirrhotic ascites, 18 patients from the inpatient setting and 7 patients from the outpatient setting were enrolled (Figure 2). The 25 enrolled patients had a mean age of 58 years (SD 13; range 35-81 years), mean model for end-stage liver disease (MELD) score of 15.8 (SD 5.9), and 12 (48%) were men. The etiology of cirrhosis was alcohol-related in 11 (44%), nonalcoholic steatohepatitis in 9 (36%), and viral infection in 3 (12%). Of the 25 patients, 5 (20%) patients were on 1 diuretic at enrollment, 17 (68%) were on 2 diuretics, and 3 (12%) were on no diuretics (Table 1).

**Table 1:** Patient characteristics and enrollment.

Patient characteristics		Total cohort (N=25)	Inpatient enrollees (n=18)	Outpatient enrollees (n=7)	*P* value^a^
Age (years), mean (SD)		57.6 (12.8)	60.1 (9.9)	51.3 (17.6)	.12
Male, n (%)		48	39	71	.20
**Etiology of cirrhosis, n (%)**					
	Alcohol	44	44	43	.91
	Nonalcoholic steatohepatitis	36	39	29	.91
	Viral	12	11	14	.91
	Other	8	6	14	.91
Model for end-stage liver disease, mean (SD)		15.8 (5.9)	16.7 (6.5)	13.3 (2.9)	.20
**Diuretics, n (%)**					
	Furosemide alone	12	11	14	.80
	Spironolactone alone	8	11	0	.80
	Furosemide and spironolactone	68	61	86	.80
	None	12	17	0	.80
Diagnostic paracentesis during admission, n (%)		N/A^b^	61	N/A	N/A
Therapeutic paracentesis during admission, n (%)		N/A	50	N/A	N/A

^a^Comparing inpatient and outpatient subgroups.

^b^N/A: not applicable.

### Weight Data Transmission

Patients were enrolled in the ascites monitoring program for 979 total patient-days (Table 2). Weight data were successfully transmitted into the EMR on 697 (71.1%) days. On 162 days (16.5% of the enrollment days), weight data did not appear in the EMR, and patients reported a likely issue with technology. The remote nature of this intervention limited the ability of study staff to precisely ascertain the cause of each technology issue, but the most common culprits included the scale not pairing to the phone, weight data not transmitting from the phone into the EMR, and spontaneous disconnections of Bluetooth or the internet. Updates were made to the app to improve data transmission from older smartphone operating systems, which reduced technology issues over the course of the study.

Patients were more likely to weigh themselves in the morning, with 593 weights transmitted before noon, as opposed to 104 transmitted after noon. The percentage of days with weight data in the morning was not associated with phone calls (*P*=.33), technology issues (*P*=.46), alerts fired (*P=*.58), or admissions (*P=*.96).

The location of enrollment, inpatient or outpatient, did not lead to differences in the number of days with weight data transmitted (*P*=.29) or the number of calls required (*P*=.51).

**Table 2:** Smartphone app outcomes by the patient.

Patient	Days enrolled	Days with weight in EMR^a^	Calls for no weight in EMR	Days of technology issues	Weights before/after noon	Alerts fired	Weight increase alerts	Weight decrease alerts	Provider responded to alert	Provider intervened after alert	Admissions while enrolled
1	30	10	13	21	6/4	1	0	1	1	0	1
2	25	25	0	0	13/12	4	0	4	3	2	1
3	28	27	1	2	25/2	2	2	0	2	1	0
4	34	25	7	0	24/1	0	0	0	0	0	1
5	32	28	1	1	27/1	1	0	1	0	0	1
6	45	27	4	0	26/1	2	2	0	2	2	6
7	16	9	7	7	3/6	1	1	0	1	1	0
8	12	7	0	0	3/4	2	1	1	1	1	1
9	28	28	0	0	28/0	1	0	1	1	1	0
10	43	29	4	0	28/1	3	2	1	3	2	0
11	44	33	3	0	17/16	0	0	0	0	0	0
12	35	33	1	0	32/1	1	1	0	0	0	2
13	62	25	5	32	25/0	1	0	1	1	1	0
14	51	3	9	45	3/0	0	0	0	0	0	0
15	61	19	7	37	17/2	3	1	2	2	1	2
16	42	42	0	0	42/0	8	5	3	8	3	1
17	28	10	5	3	8/2	2	1	1	2	2	0
18	72	67	1	0	63/4	16	9	7	12	7	0
19	87	80	7	0	53/27	13	8	5	11	11	0
20	27	27	0	0	27/0	6	5	1	5	3	0
21	43	28	7	9	22/6	2	1	1	2	0	0
22	13	3	5	0	2/1	0	0	0	0	0	1
23	58	54	3	2	46/8	3	2	1	3	1	0
24	28	26	0	0	23/3	4	0	4	3	3	0
25	35	32	2	3	30/2	3	0	3	3	3	0
Total, n (%)^b^	979 (100)	697 (71.1)	92 (9.3)	162 (16.5)	593/104 (60.5/10.6)	79 (8.0)	41 (4.1)	38 (3.8)	66 (6.7)	45 (4.5)	17^c^

^a^EMR: electronic medical record.

^b^Percentage of event occurrence per the total number of days enrolled.

^c^Not applicable.

### Weight Change Alerts

There were 79 weight alerts emailed to hepatology providers during the study period, meaning that a notable weight change occurred on 8.0% (79/979) of the days that patients were enrolled in the program. The weight alerts were evenly divided between alerts for weight increase (41/79 alerts, 52%) and weight decrease (38/79 alerts, 48%). Of the 25 patients, 10 (40%) patients prompted both weight increase and weight decrease alerts during the study period, 7 (28%) patients prompted only weight decrease alerts, 4 (16%) patients prompted only weight increase alerts, and 4 (16%) patients prompted no alerts.

**Figure 3:**
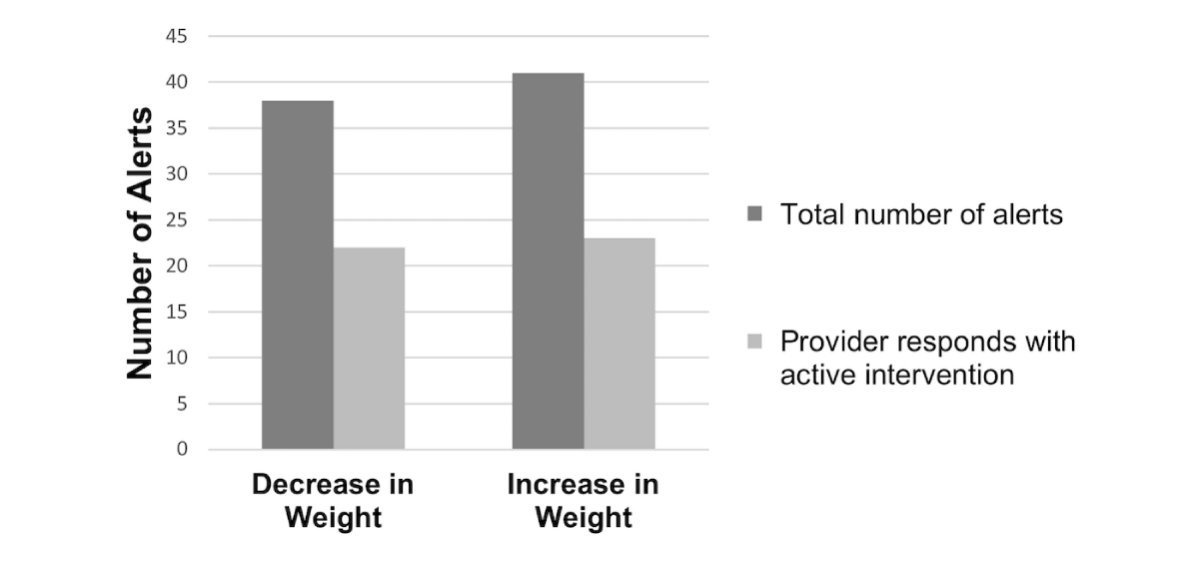
Provider response by weight alert type (*P*=.87).

Providers responded in some fashion to 66 of the 79 (84%) alerts and responded with an active intervention to 45 (57%) alerts. Active interventions included communicating with the patient, ordering testing, adjusting diuretics, ordering a paracentesis, or scheduling a follow-up appointment. Provider responses not characterized as *active intervention* included an acknowledgment of the alert and, in many cases, an explanation as to why the weight change was expected. Providers were equally likely to intervene on a weight increase alert as they were to a weight decrease alert (Figure 3; *P*=.87).

For every weight increase alert, patients were asked by the study staff about shortness of breath, early satiety, and a tense abdomen. For every weight decrease alert, patients were asked about dizziness and nausea, vomiting, or diarrhea. For all alerts, patients were queried about diuretic compliance. For 24 of the 79 weight alerts, the patient could not be reached on that day to report symptoms. With weight increase alerts, 14 of the 28 patients (50%) reported shortness of breath, 12 reported (43%) early satiety, and 14 (50%) reported a tense abdomen. With 27 weight decrease alerts, 8 (30%) reported dizziness and 10 (37%) reported nausea, vomiting, or diarrhea. Patients reported a diuretic compliance 84% (46/55) of the time. In the Fisher exact test, reporting shortness of breath (*P*=.16), early satiety (*P*=.09), tense abdomen (*P*=.06), dizziness (*P*=.83), and nausea, vomiting, or diarrhea (*P*=.75) were not associated with the nature of provider response. Compliance with diuretics was also not associated with the nature of the provider response (*P*=.07).

Patients underwent a total of 13 paracenteses during the study period. Of the 38 weight decrease alerts, 10 (26%) occurred within 2 days of a paracentesis. Of the 41 weight increase alerts, 15 (37%) were followed by a referral for paracentesis within 7 days.

### Engagement

A total of 92 phone calls were made by the study staff when weight data did not appear in the EMR, with a mean of 3.7 calls (SD 3.5) per patient and a range of 0 to 13 calls per patient. The number of phone calls correlated with days of technology issues (*r*=0.60; *P*=.002), perhaps because calls were prompted by a lack of weight data in the EMR. The number of phone calls was not correlated with the number of hospital admissions during the study period (*P*=.88), MELD score (*P*=.59), or the number of weight alerts (*P*=.27).

**Figure 4:**
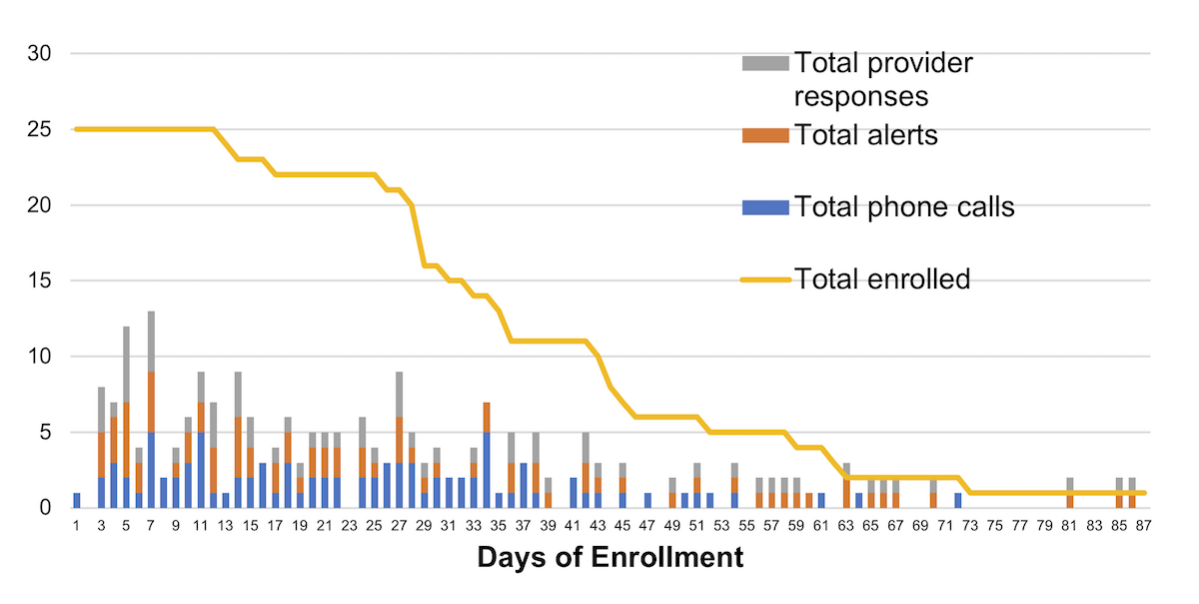
Calls and alert responsiveness during enrollment.

Providers responded to 84% (66/79) of the alerts, with a response rate range of 0% to 100%. There was no trend of providers responding to alerts more frequently at the beginning of enrollment as compared with at the end of enrollment (Figure 4). There were 12 hepatology providers with patients enrolled in this program, including 7 attendings, 3 fellows, and 2 nurse practitioners. Nurse practitioners and fellows work with attendings but were the primary responders to alerts in this program. Using the Fisher exact test, we found a significant difference in responsiveness to alerts by provider type, with fellows performing an active intervention to 75% (24/32) of the alerts, nurse practitioners to 59% (10/17) of the alerts, and attendings to 37% (11/30) of the alerts (*P*=.03).

By default, patients were enrolled for 28 days but could remain in the program longer if both the patient and the hepatology provider desired longer enrollment. Out of the 25 patients, 15 (60%) patients extended their participation for over 30 days, and 6 of those 15 (24%) were enrolled for over 50 days. On the other hand, 3 of the 25 (12%) patients chose to withdraw from the program within 25 days. The reasons for early withdrawal included technology issues, being too ill to stand on the scale, and being nonresponsive to phone calls.

### Patient, Caregiver, and Hepatology Provider Feedback

Eighteen patients, 10 hepatology providers, and 4 caregivers provided a detailed exit interview. Patients felt that the program was easy (17/18, 94%), looked at their weights in the smartphone app (15/18, 88%), and preferred the smartphone app to another digital tool such as a computer (94%). Patients and caregivers found benefits of the program to increase connectedness to providers, a better sense of ascites status, ease of the program, and increased adherence to weight tracking at home (Table 3 and Textbox 1). Patients and caregivers who experienced technology issues were frustrated by those problems, yet some still found the overall program beneficial.

**Table 3:** Patient and caregiver exit interview responses: quantitative results from patient exit interviews (n=18).

Questions^a^		Response, n (%)	Total, N
**Facilitators and barriers**			
	Was the program easy?	Yes, 17 (94)	18
	Was it difficult to remember daily weights?	Yes, 2 (11)	18
	Did you look at the app during the program?	Yes, 15 (88)	17
	Did you have technical difficulties?	Yes, 8 (44)	18
**Root causes**			
	Can you name your diuretics?	Yes, 11 (65)	17
	In the last week, have you missed your diuretics?	Yes, 2 (12)	16
	Have you had difficulty paying for medications?	Yes, 2 (12)	16
	Is weight monitoring important for ascites management?	Yes, 18 (100)	16
	Have you consumed alcohol recently?	Yes, 1 (6)	17
	Will you stay somewhere else in the next month?	Yes, 2 (12)	16
**Desires features of the ultimate digital tool**			
	Do you prefer another device type (eg, computer) over a smartphone?	Yes, 1 (6)	18
	Was the timing of phone calls a problem?	Yes, 0 (0)	18

^a^Abbreviated for ease of interpretation. For the full interview script, see Multimedia Appendix 1.

**Themes and representative quotes from patient and caregiver exit interviews.**

Benefit of program: connectedness to providers

- “I just feel better knowing that my doctor is aware of my weights on a daily basis.”
- “It was awesome in that I was in constant communication with my doctor about what was going on in terms of my weight and how to proceed. Do we need more diuretics … do we need a paracentesis?”

Benefit of program: better sense of ascites status

- “If I leave it to memory, I only remember yesterday’s [weight]. The program gives me a whole history, so I can look back 5 days, 7 days, to see if there were any real fluctuations.”
- “It made me be more aware of my sodium intake.”

Ease of program

- “I really liked that it was something I could do every morning, and I could see the ascites was going away.”

Other benefits

- “Where before I might have had 90% compliance, now I have 100% compliance.”
- “I don’t think that she would’ve had all the problems that she’s had, if she would’ve had this scale a long time ago. I mean, it seems like a simple thing, but for someone with this problem, it’s a huge deciding factor. It really is.”

Challenges

- “It was kind of difficult for me because I’m not very savvy on cellphones.”
- “Was a great idea and all that, but it’s very frustrating when you try to set it up and it doesn’t work.”

Root causes of ascites mismanagement

- “They told me that if I have an uncomfortable feeling, a hard stomach or difficulty breathing, [I should] call them to make an appointment. The only problem I had with that is I don’t know which doctor to get in touch with.”
- “He’s the problem… trying to keep him away from salt and especially processed meats.”
- “I’ve been out of work… Sometimes I go without my medications a lot.”

Desired features of future tool

- “Phone app is good for me.”
- “I prefer something of this nature on my laptop.”

Hepatology providers generally found the program easy and helpful (Textbox 2). They enjoyed regular access to accurate weight data, the content of weight alerts, and the dialogue the program created between the provider and the patient. Many providers noted a small increase in work required to respond to weight alerts, but most did not find this burdensome. A few providers described features of the ideal patient to enroll in this program: ascites is symptomatic, the patient is motivated to engage with the medical system and improve health, and the patient is relatively compensated medically outside of ascites (see Multimedia Appendix 2 for expanded quotes).

**Themes and representative quotes from hepatology provider exit interviews.**

Positives of the program

- “I like having access to the weight measurements on a regular basis. It’s a lot easier than asking him to weigh himself and transmit it back to me. It definitely changed the clinical management.”
- “It allows you to keep people out of the hospital.”

Challenges of the program

- “It seemed like a number of the folks that I had, there were these weird technical issues with the scale, the Bluetooth, whatever it was.”
- “If we’re doing people who are in the hospital [and] going home, their diet changes dramatically. I don’t know that capturing their weight necessarily accurately reflects their fluid status alone. I think it’s their nutritional status also.”

How the smartphone app helps patients

- “XX is a patient who is completely new to ascites… and was just starting on diuretics. The weight tracking program actually helped her see the progress the diuretics were making… The program actually allowed us to have a dialogue about how she was supposed to lose weight.”

Features of the ideal patient enrollee

- “I think it may work better for just outpatient. And maybe starting off with a cohort that’s less sick… I think that it may be more beneficial in patients who you’re just starting on diuretics and patients who have kind of stable nutritional status, stable—not the truly decompensated cirrhotics.”
- “XX had a particularly unstable weight. His ascites was highly symptomatic. He lived a relatively long distance from the hospital. And not only did it provide us useful information, he personally liked the idea of being engaged and it gave him some sense of control over his own care and body.”

Desired features of future program

- “I think because I had multiple patients involved, the emails, occasionally, it seemed like they were coming frequently but I think that’s just because I would get a separate email per patient. So, I think if this was to be potentially rolled out and people had multiple patients involved, if they could maybe be grouped but I don’t know whether that’s possible.”

### Hospital Admissions During the Study Period

The 25 patients enrolled in this program were admitted on 17 occasions during the study period. Of the 17 admissions, 4 (24%) were related to ascites or ascites treatment, and 12% (3/25) of patients had an ascites-related admission while enrolled. A total of 4 (24%) admissions were for gastrointestinal bleeding or anemia, 3 (18%) for infection, 2 (12%) for hepatic encephalopathy, and 4 (24%) for reasons unrelated to liver disease. Of the 17 admissions, 5 (29%) occurred within 7 days after a weight alert, 15 (88%) were among inpatient enrollees, and 2 (12%) were among outpatient enrollees.

## Discussion

### Principal Findings

A simple telemonitoring system for patients with cirrhotic ascites was able to transmit weight data into the EMR on >50% of the days, and hepatology providers responded to >50% of the weight alerts, thus meeting our predetermined threshold for feasibility. Our system of a Bluetooth-connected scale and a smartphone app transmitted weight data into the EMR on 71.2% (697/979) of the days that patients were enrolled. Providers responded in some fashion to 84% (66/79) of weight change alerts and responded actively with an intervention to 57% (45/79) of alerts.

Weight change alerts appeared to correlate with ascites and influenced ascites management. Approximately one-third of the weight increase alerts were followed by an LVP. Providers responded equally to weight increase and weight decrease alerts and did not appear to respond less frequently over the course of patient enrollment. It is notable that symptoms reported with the alert did not significantly influence the nature of the provider’s response. A larger prospective study will be needed to further evaluate their utility. Only 12% (3/25) of patients were readmitted for ascites while enrolled in this program. More experience with the system or refinement of the alert criteria may improve effectiveness. Although there was no comparison group in this study, other cohorts have found 13.8% of such patients readmitted within 30 days and 25% within 90 days [2,19].

Patients and providers remained engaged throughout the program. Patients continued to transmit weight data, even at the end of their enrollment. In fact, 60% (15/25) of the patients extended their participation beyond 30 days and 24% (6/25) beyond 50 days. A few patients terminated the program early, but mainly because they became too ill to participate. Similarly, providers continued to respond to alerts throughout the enrollment period. Weight change alerts were fired on only 8% of the days that patients were enrolled; this low alert frequency likely contributed to the high rate of provider responsiveness. Finally, we were able to stop proactively screening the inpatient census, because of the increasing provider referrals during the study.

The main perceived benefits by patients and caregivers were increased connectedness to providers and a better sense of ascites status. Hepatology providers likewise enjoyed the easy dialogue with patients facilitated by the program.

### Limitations

Technology issues impaired weight data transmission, provoked phone calls, and impacted patient experience. Patients reported some form of technology issue on 16.5% (162/979) of the days enrolled. At times, this involved an unpaired scale and a smartphone app, connectivity problems, or the need for a software update. Some of these issues were resolved with software updates, and technology issues decreased over the course of the study. Patients and caregivers who experienced technology issues expressed frustrations in their exit interviews, yet most still found the overall program beneficial.

Hepatology provider interviews revealed that certain patients may benefit from this program more than others. They described the ideal enrollee as having symptomatic ascites, motivated to engage with the medical system and improve health, and be relatively compensated outside of their ascites. Although patients with other active medical issues, such as gastrointestinal bleeding or malnutrition, may be at higher risk for poor outcomes, their other medical issues may influence their weight and therefore the efficacy of this program.

We suspect that there are several reasons why this telemonitoring program was feasible at our center. First, we had access to a smartphone app that was able to securely transmit weight data from the patient’s home into our EMR. Second, the app allowed both patients and providers to be immediately aware of accurate weight trends. Third, the program was at no cost to the patients. Fourth, hepatology providers needed to exert little effort to enroll their patients, and the program generated high-yield information (weight alerts) for their attention, on only 8.1% (79/979) of the days that patients were enrolled. Finally, the telemonitoring program directly addressed a core challenge of ascites management: lack of accurate, timely weight data reaching hepatology providers.

Telemonitoring programs are not a stand-alone solution for ascites management. The program required study staff to monitor weights, make phone calls, formulate alerts, facilitate easy enrollment, and ask field questions. We suspect that the program would not have been feasible without this larger infrastructure surrounding the app.

Rigorous evidence supporting the efficacy of mobile health interventions in chronic disease is lacking [20]. Future studies on telemonitoring interventions should be based on lessons learned in feasibility studies such as this one, assessing efficacy using validated methods, and assessing the cost-effectiveness of performing such interventions on a larger scale [21].

### Conclusions

A smartphone-based telemonitoring system was feasible for the management of cirrhotic ascites. Future studies are required to assess the efficacy of such a program in reducing hospital admissions and improving patient and provider experience.

## Appendix

Multimedia Appendix 1 Exit interviews.

Multimedia Appendix 2 Expanded quotes.

## Abbreviations

EMR: electronic medical record
LVP: large-volume paracentesis
MELD: model for end-stage liver disease
PGHDConnect: patient-generated health data connect

## References

[1] Macdonald S, Jepsen P, Alrubaiy L, Watson H, Vilstrup H, Jalan R (2019). Quality of life measures predict mortality in patients with cirrhosis and severe ascites. Aliment Pharmacol Ther.

[2] Tapper EB, Halbert B, Mellinger J (2016). Rates of and reasons for hospital readmissions in patients with cirrhosis: a multistate population-based cohort study. Clin Gastroenterol Hepatol.

[3] Scaglione SJ, Metcalfe L, Kliethermes S, Vasilyev I, Tsang R, Caines A, Mumtaz S, Goyal V, Khalid A, Shoham D, Markossian T, Luke A, Underwood H, Cotler SJ (2017). Early hospital readmissions and mortality in patients with decompensated cirrhosis enrolled in a large national health insurance administrative database. J Clin Gastroenterol.

[4] Shaheen A, Nguyen HH, Congly SE, Kaplan GG, Swain MG (2019). Nationwide estimates and risk factors of hospital readmission in patients with cirrhosis in the United States. Liver Int.

[5] di Pascoli M, Ceranto E, de Nardi P, Donato D, Gatta A, Angeli P, Pontisso P (2017). Hospitalizations due to cirrhosis: clinical aspects in a large cohort of Italian patients and cost analysis report. Dig Dis.

[6] Volk ML, Tocco RS, Bazick J, Rakoski MO, Lok AS (2012). Hospital readmissions among patients with decompensated cirrhosis. Am J Gastroenterol.

[7] Planas R, Montoliu S, Ballesté B, Rivera M, Miquel M, Masnou H, Galeras JA, Giménez MD, Santos J, Cirera I, Morillas RM, Coll S, Solà R (2006). Natural history of patients hospitalized for management of cirrhotic ascites. Clin Gastroenterol Hepatol.

[8] Runyon BA (2013). Introduction to the revised American association for the study of liver diseases practice guideline management of adult patients with ascites due to cirrhosis 2012. Hepatology.

[9] European Association for the Study of the Liver (2010). EASL clinical practice guidelines on the management of ascites, spontaneous bacterial peritonitis, and hepatorenal syndrome in cirrhosis. J Hepatol.

[10] Tapper E, Baki J, Hummel S, Lok A (2019). Design and rationale of a randomized-controlled trial of home-delivered meals for the management of symptomatic ascites: the SALTYFOOD trial. Gastroenterol Rep (Oxf).

[11] Bellos I, Kontzoglou K, Psyrri A, Pergialiotis V (2020). Tolvaptan response improves overall survival in patients with refractory ascites: a meta-analysis. Dig Dis.

[12] Ginès P, Wong F, Watson H, Milutinovic S, del Arbol LR, Olteanu D, HypoCAT Study Investigators (2008). Effects of satavaptan, a selective vasopressin V(2) receptor antagonist, on ascites and serum sodium in cirrhosis with hyponatremia: a randomized trial. Hepatology.

[13] Bloom PP, Marx M, Wang TJ, Green B, Ha J, Bay C, Chung RT, Richter JM (2020). Attitudes towards digital health tools for outpatient cirrhosis management in patients with decompensated cirrhosis. BMJ Innov.

[14] Siddique SM, Lane-Fall M, McConnell MJ, Jakhete N, Crismale J, Porges S, Khungar V, Mehta SJ, Goldberg D, Li Z, Schiano T, Regan L, Orloski C, Shea JA (2018). Exploring opportunities to prevent cirrhosis admissions in the emergency department: a multicenter multidisciplinary survey. Hepatol Commun.

[15] Hudson B, Round J, Georgeson B, Pring A, Forbes K, McCune CA, Verne J (2018). Cirrhosis with ascites in the last year of life: a nationwide analysis of factors shaping costs, health-care use, and place of death in England. Lancet Gastroenterol Hepatol.

[16] Malterud K (2001). Qualitative research: standards, challenges, and guidelines. Lancet.

[17] Giacomini MK, Cook DJ (2000). Users' guides to the medical literature: XXIII. Qualitative research in health care B. What are the results and how do they help me care for my patients? Evidence-based medicine working group. J Am Med Assoc.

[18] Bradley EH, Curry LA, Devers KJ (2007). Qualitative data analysis for health services research: developing taxonomy, themes, and theory. Health Serv Res.

[19] Seraj SM, Campbell EJ, Argyropoulos SK, Wegermann K, Chung RT, Richter JM (2017). Hospital readmissions in decompensated cirrhotics: factors pointing toward a prevention strategy. World J Gastroenterol.

[20] Bashi N, Fatehi F, Fallah M, Walters D, Karunanithi M (2018). Self-management education through mhealth: review of strategies and structures. JMIR Mhealth Uhealth.

[21] Marcolino MS, Oliveira JA, D'Agostino M, Ribeiro AL, Alkmim MB, Novillo-Ortiz D (2018). The impact of mhealth interventions: systematic review of systematic reviews. JMIR Mhealth Uhealth.

