# Supplementary material for: A Smartphone App to Manage Cirrhotic Ascites Among Outpatients: Feasibility Study
Source: JMIR Med Inform. 2020 Sep 2;8(9):e17770. doi: 10.2196/17770 (PMC7495260; doi:10.2196/17770)
Supplement: Multimedia Appendix 1 [file medinform_v8i9e17770_app1.docx]

**Supplementary Appendix**

**Patient Exit Interview**

*Facilitators and Barriers*

1. What did you like about this weight tracking program?
2. What parts of the study were easy to do?
3. What did you dislike about the weight tracking program?
4. What parts of the study were hard to do?
5. If not mentioned: was it difficult to remember to weight yourself every day?
6. If not mentioned: did you use the Smartphone application to look at your weights?
7. If not mentioned: was the Smartphone application easy or difficult to use?
8. If not mentioned: did you have technical difficulties with the app, scale, or the internet?
9. If not mentioned: what did you think of the phone calls from study staff members?

*Explore root causes: (Diet questions adopted from USDA’s Automated Multiple-Pass Method)*

1. Did medical staff explain how to treat your ascites when you were admitted to the hospital or in an office visit? What did they say?
   1. If so, what did you like about that education?
   2. What more do you wish you learned?
2. Which medication(s) do you take to treat the ascites?
3. In the last week, how many days did you miss taking your diuretics (ascites medications)?
4. Have you had any doctor visits since being discharged or since starting this program?
5. Can you tell me all the foods you ate yesterday (or pick a more typical day recently)? (cues: yesterday, midnight to midnight, activities, snacks and beverages, location)
   1. Were there any other beverages, alcoholic beverages, sweets, savory snacks, fruit, vegetables, cheeses, breads or rolls?
   2. Did you eat a breakfast? Lunch? Dinner? Snack?
   3. Clarify each meal content and time (for example: “for breakfast you had a bagel and coffee, did you have anything else?” “Did you have anything between your 7am breakfast and your 10am snack?”)
   4. Finally, any other foods outside of meals we might be forgetting? (probe: in the car, at the mall, walking around, at meeting, while cooking, from a friend).
6. Have you had difficulty being able to pay for your medications in the last couple months?
7. Do you believe that weight monitoring is important to managing your ascites? Why or why not?
8. Have you consumed alcoholic beverages since discharge? If so, approximately how many?
9. Have you been living in one place in the last couple months?
10. Do you think you may be staying somewhere else in the next month?

*Explore design features of the ultimate digital tool:*

1. How did you feel about the program using a Smartphone application? Would you have preferred something else (website, computer application, phone calls only, texts only)?
2. Was the timing of this system (when you weighed yourself, when study staff called) an issue?
3. Is there anything else you would add to this system to make it work better for you?

**Caregiver Exit Interview**

*Facilitators and Barriers*

1. What did you like about the weight tracking program your loved one participated in?
2. What parts of the study were easy to do?
3. What did you dislike about the weight tracking program your loved one participated in?
4. What parts of the study were hard to do?
5. If not mentioned: was it difficult for them to remember to weight themselves every day?
6. If not mentioned: did you use the Smartphone application to look at their weights?
7. If not mentioned: was the Smartphone application easy or difficult to use?
8. If not mentioned: did you have technical difficulties with the application, scale, or the internet?
9. If not mentioned: what did you think of the phone calls from study staff members?

*Explore root causes:*

1. Has medical staff explain how to treat your loved one’s ascites? What did they say?
   1. If so, what did you like about that education?
   2. What more do you wish you learned?
2. In the last week, how many days did your loved one miss taking his or her diuretics (ascites medications)?
3. Does your loved one follow a strict low salt diet?
4. Have you and your loved one had difficulty being able to pay for medications recently?
5. Do you believe that weight monitoring is important to managing her or her ascites? Why or why not?

*Explore design features of the ultimate digital tool:*

1. How did you feel about the program using a Smartphone application? Would you have preferred something else (website, computer application, phone calls only, texts only)?
2. Was the timing of this system (when they needed to weigh themselves, when study staff called) an issue?
3. Is there anything else you would add to this system to make it work better for you and your loved one?

**Hepatologist Exit Interview**

*Facilitators and Barriers*

1. What did you like about the outpatient weight tracking program your patient participated in?
2. What parts of the program were easy to do?
3. What did you dislike about the weight tracking program?
4. What parts of the program were hard to do?
5. Do you think your patient benefited from being in the program?
6. If not mentioned: did you find the weight alerts helpful?
7. If not mentioned: how would you change the content or timing of the weight reports and alerts?

*Explore root causes:*

1. What did you hope the outcome of this program would be for your patient? Did they achieve that? Why or why not?
2. Did you learn new information from this study (ie weight alerts, etc) that you would not have otherwise learned?
3. If so: was that useful information?

*Explore design features of the ultimate digital tool:*

1. How did you feel about the program sending you emails with weight reports? Would you have preferred another mode (ex: EPIC inbox reports)?
2. Did you receive weight reports and alerts at appropriate times? If not, how would you improve the timing?
3. What else would you change to this system to make it work better for you?
