# Supplementary material for: A Smartphone App to Manage Cirrhotic Ascites Among Outpatients: Feasibility Study
Source: JMIR Med Inform. 2020 Sep 2;8(9):e17770. doi: 10.2196/17770 (PMC7495260; doi:10.2196/17770)
Supplement: Multimedia Appendix 2 [file medinform_v8i9e17770_app2.docx]

**Supplementary Appendix 2: Expanded Quotes**

| **Theme** | **Representative Quotes from Patients and Caregivers** |
| --- | --- |
| *Benefit of Program: Connectedness to Providers* | “I just feel better knowing that my doctor is aware of my weights on a daily basis.”  “It was awesome in that I was in constant communication with my doctor about what was going on in terms of my weight and how to proceed. Do we need more diuretics… do we need a paracentesis?”  “It was… a good sense of security knowing that somebody was kind of watching it on the other end.” |
| *Benefit of Program: Better Sense of Ascites Status* | “I like this one because it’s right there on my phone… My health care proxy… will always ask ‘How’s your weight today?’… I can look back at the week and see what happened.”  “If I leave it to memory, I only remember yesterday’s [weight]. The program gives me a whole history, so I can look back five days, seven days, to see if there were any real fluctuations.”  “It made me be more aware of my sodium intake.” |
| *Ease of Program* | “The scale? I loved it. I love the app on my phone. Very easy and I still do it every day.”  “I really liked that it was something I could do every morning, and I could see the ascites was going away.” |
| *Other Benefits* | “Where before I might have had 90% compliance, now I have 100% compliance”  “I don’t think that she would’ve had all the problems that she’s had, if she would’ve had this scale a long time ago. I mean, it seems like a simple thing, but for someone with this problem, it’s a huge deciding factor. It really is.” |
| *Challenges* | “I liked the study itself… Only that it’s very frustrating when you spend half an hour trying to get it to work and it doesn’t work.”  “It was kind of difficult for me because I’m not very savvy on cellphones.”  “Was a great idea and all that, but it’s very frustrating when you try to set it up and it doesn’t work.” |
| *Root Causes of Ascites Mis-Management* | “They told me that if I have an uncomfortable feeling, a hard stomach, difficulty breathing to call them, make an appointment. The only problem I had with that is: I don’t know which doctor to get in touch with.”  “I was informed yesterday that I was being discharged (from rehab) on Monday and I have no place to go. I’m homeless.”  Do you remember the ascites education you received in the hospital? “Not really. I was kind of out of it.”  “He’s the problem… trying to keep him away from salt and especially processed meats.”  “I’ve been out of work… Sometimes I go without my medications a lot.” |
| *Desired Features of Future Tool* | “Phone app is good for me.”  “Tell you the truth… I don’t really go on Smartphones and I don’t go on the computer.”  “I prefer something of this nature on my laptop.” |

| **Theme** | **Representative Quotes from Hepatology Providers** |
| --- | --- |
| *Positives of the Program* | “I like having access to the weight measurements on a regular basis. It’s a lot easier than asking him to weigh himself and transmit it back to me. It definitely changed the clinical management.”  “It allows you to keep people out of the hospital.”  “I found the weight alerts exceedingly helpful. They contained exactly the information I wanted to know.” |
| *Challenges of the Program* | “Maybe when I was getting a lot of emails, it was annoying, but it was actually very helpful.”  “It seemed like a number of the folks that I had, there were these weird technical issues with the scale, the Bluetooth, whatever it was.”  “If we’re doing people who are in the hospital [and] going home, their diet changes dramatically. I don’t know that capturing their weight necessarily accurately reflects their fluid status alone. I think it’s their nutritional status also.” |
| *How the Smartphone Application Helps Patients* | “XX is a patient who is completely new to ascites… and was just starting on diuretics. The weight tracking program actually helped her see the progress the diuretics were making… The program actually allowed us to have a dialogue about how she was supposed to lose weight.”  “I think that the outcome of his care was enhanced a little bit by [the program], but his perception of his care was enhanced a lot.” |
| *Features of the Ideal Patient Enrollee* | “I think it may work better for just outpatient. And maybe starting off with a cohort that’s less sick… I think that it may be more beneficial in patients who you’re just starting on diuretics and patients who have kind of stable nutritional status, stable-- not the truly decompensated cirrhotics.”  “XX had a particularly unstable weight. His ascites was highly symptomatic. He lived a relatively long distance from the hospital. And not only did it provide us useful information, he personally liked the idea of being engaged and it gave him some sense of control over his own care and body.” |
| *Desired Features of Future Program* | “I think because I had multiple patients involved, the emails, occasionally, it seemed like they were coming frequently but I think that’s just because I would get a separate email per patient. So, I think if this was to be potentially rolled out and people had multiple patients involved, if they could maybe be grouped but I don’t know if that’s possible.”  “These people are really sick and so it’s hard for me to envision kind of true alert fatigue without - on the other hand alert fatigue does exist in general. And so we might be able to make the algorithm somewhat more subtle and eliminate the weight loss alerts after a paracentesis.” |
